# Older Adults' Self‐Care and Family Caregiver Contribution in Multiple Chronic Conditions: A Dyadic Qualitative Study

**DOI:** 10.1111/jan.70246

**Published:** 2025-10-05

**Authors:** Giulia Andrea Baldan, Irene Dello Iacono, Valentina Giurissevich, Davide Ausili, Ercole Vellone, Maddalena De Maria, Maria Matarese

**Affiliations:** ^1^ Department of Biomedicine and Prevention University of Rome Tor Vergata Rome Italy; ^2^ Health Professional Management Service (DPS) University Hospital of Padua Padua Italy; ^3^ Department of Medicine and Surgery University of Milan—Bicocca Monza Italy; ^4^ Department of Nursing and Obstetrics Wroclaw Medical University Wroclaw Poland; ^5^ Department of Life Health Sciences and Health Professions Link Campus University Rome Italy; ^6^ Research Unit of Nursing Sciences, Faculty of Medicine and Surgery Campus Bio‐Medico University of Rome Rome Italy

**Keywords:** caregiving, chronic illness, decision‐making, older people, self‐care

## Abstract

**Aims:**

To explore how older adult‐family caregiver dyads jointly manage multiple chronic conditions. Specifically, it investigates how dyads (i) prioritise chronic diseases, (ii) make and negotiate decisions related to self‐care and (iii) define and distribute self‐care tasks and caregiver contributions.

**Design:**

A qualitative descriptive study using dyadic data collection and analysis.

**Methods:**

Semi‐structured interviews were conducted separately with chronically ill older adults and their family caregivers between July and December 2024. A hybrid inductive‐deductive content analysis was applied. Dyadic analysis compared intra‐dyad perspectives to identify patterns of agreement and disagreement.

**Results:**

Thirty‐four dyads (*n* = 68 participants) were interviewed. Older adults had a mean age of 80.09 years (SD = 6.95) and were affected by a median of four chronic conditions. Family caregivers had a mean age of 51.71 years (SD = 14.59), with most being the older adults' children (66.67%) and women (82.35%). Five categories, comprising 25 subcategories, were derived from the data. Disease prioritisation varied within dyads: older adults often focused on conditions with the most disabling symptoms, while caregivers emphasised those with higher risks of complication. Decision‐making roles ranged from older adult‐led to caregiver‐led to shared. Care organisation followed three models: collaborative, older adult‐directed, or caregiver‐directed. Challenges in managing diseases included treatment adherence, care coordination, emotional burden and addressing multiple symptoms simultaneously. Role distribution in disease management and decision‐making was complex and occasionally misaligned, sometimes resulting in conflict. Collaborative dyads reported greater adaptability and balance, while incongruent dyads experienced relational and organisational strain.

**Conclusion:**

Managing multiple chronic conditions in older adults is a relational process shaped by interpersonal dynamics and shared responsibilities with family caregivers. Recognising dyadic relational patterns is essential for designing targeted educational interventions. Nurses should incorporate dyadic assessments into routine care to improve outcomes for older adults and reduce caregiver burden.

**Implications for the Profession and/or Patient Care:**

This study highlights the importance of viewing chronic disease management as a dyadic process, rather than an individual task, involving both the older adult and the family caregiver. Tailored strategies that account for the relational dynamics within dyads, such as decision‐making roles and care task distribution, are essential for effective chronic disease management.

**Reporting Method:**

Consolidated criteria for reporting qualitative studies (COREQ).

**Patient or Public Contribution:**

None.

**Summary**

- What problem did the study address?
  ○ The study examined how older adults with multiple chronic conditions and their informal caregivers jointly manage self‐care, specifically how they prioritise diseases, make decisions and share care responsibilities.
- What were the main findings?
  ○ Older adult–family caregiver dyads adopt different models of disease management (collaborative, older adult‐directed, caregiver‐directed), often holding divergent views on disease importance and decision‐making roles, and facing both shared and individual challenges. Misalignment in roles can lead to strain, whereas congruent dyads tend to demonstrate greater adaptability.
- Where and on whom will the research have an impact?
  ○ The findings have implications for nurses caring for older adults with multiple chronic conditions and their family caregivers, guiding the development of personalised, dyad‐centred interventions. They are relevant to clinical settings, home care, and caregiver support programmes, particularly within ageing populations managing multiple chronic conditions.
- What does this paper contribute to the wider global clinical community?
  ○ Nurses should shift from an individual‐centric to a relational model of care to better assess and support older adult–caregiver dyads in managing multiple chronic conditions.
  ○ Distinct patterns of care organisation and decision‐making roles within dyads should inform tailored interventions aimed at improving older adults' health outcomes and reducing family caregiver burden.
  ○ Dyadic, relationship‐centred approaches in chronic disease management are essential and relevant across diverse cultural and healthcare contexts.

## 1 Introduction

The global population is aging, with the proportion of individuals aged 60 and over expected to double by 2050 (WHO 2024). Most older adults live with multiple chronic conditions (MCCs), defined as the co‐occurrence of two or more chronic diseases (National Council on Aging 2024). MCCs are associated with poorer physical functioning, diminished quality of life, and worse health outcomes (Aubert et al. 2022; Makovski et al. 2019). Managing chronic conditions involves self‐care, defined as the process by which individuals with chronic illness maintain health and manage their symptoms (Riegel et al. 2012). Self‐care becomes more complex in the context of MCCs due to overlapping symptoms, competing priorities, and potential cognitive or physical limitations (Baldan et al. 2024). Effective self‐care has been shown to reduce hospitalisations and mortality rates in heart failure (HF) (Iovino et al. 2021; Jaarsma et al. 2021), improve glycaemic control and lower risk of complications in diabetes mellitus (DM) (Fabrizi et al. 2020), and reduce respiratory‐related hospital admissions in chronic obstructive pulmonary disease (COPD) (Schrijver et al. 2022).

## 2 Background

Among older adults, self‐care activities are oriented toward promoting well‐being and the prevention and management of the effects of aging, and they take place within a social and relational context (Lommi et al. 2015). Self‐care in later life is rarely carried out in isolation; it often involves the support of informal caregivers—typically family members or friends—who play an active role in sustaining these efforts (Conway 2019; Luichies et al. 2021). This support, conceptualised as the caregiver's contribution to self‐care (Vellone et al. 2019), varies in intensity and is continually negotiated within chronically ill older adult‐caregiver dyads (De Maria et al. 2021). As needs evolve, older adults and their caregivers often shift roles and renegotiate responsibilities, highlighting the relational and dynamic nature of self‐care in this population (Lommi et al. 2015).

Little is known about how older adult–informal caregiver dyads manage the complexity of MCCs. Previous research has identified four dyadic care typologies in the management of chronic diseases: patient‐oriented, where the patient manages their health independently with minimal caregiver involvement; caregiver‐oriented, where the caregiver manages the patient's health with limited patient participation; collaborative, involving active participation from both parties; and incongruent, where the patient and the caregiver disagree on who manages the illness (Buck et al. 2013). Collaborative dyads have demonstrated the highest levels of self‐care and caregiver contribution to self‐care due to more balanced task distribution (De Maria, Erba, et al. 2023; De Maria, Lee, et al. 2023). Conversely, incongruent dyads often experience conflicting expectations, which can lead to poorer health outcomes and increased caregiver burden (Irani et al. 2023). These findings highlight the importance of examining how dyadic interactions influence self‐care behaviours in the context of MCCs. Factors such as gender also shape dyadic dynamics. For example, women caregivers often excel in disease monitoring, and older patients tend to benefit more from the support provided by women caregivers (De Maria, Erba, et al. 2023; De Maria, Lee, et al. 2023).

Despite these insights, important gaps remain in understanding the lived experiences of dyads managing MCCs. Qualitative literature is limited and primarily focused on single chronic diseases. Existing studies highlight the caregiver's central role in medication adherence, symptom monitoring, exacerbation management, emotional support, and patient advocacy (Durante et al. 2019; Matarese et al. 2021). Moreover, qualitative studies also reveal the significant burden and strain that family caregivers often experience (Pendoni et al. 2024). These studies affirm the value of qualitative approaches in revealing the complex, relational aspects of disease management within dyads—aspects missed by quantitative research. A deeper understanding of how dyads face challenges, adopt self‐care strategies, and negotiate responsibilities is crucial for designing tailored interventions that enhance both older adult self‐care and caregiver contribution, ultimately improving health outcomes for older adults.

## 3 The Study

### 3.1 Aims

The aim of this study was to explore the experiences of dyads in managing MCCs, specifically investigating: (i) how older adults and caregivers, individually and jointly, establish priorities in disease management and determine which conditions to focus on; (ii) how they make decisions regarding MCC management and navigate potential conflicts; and (iii) how they define and distribute patient self‐care activities and caregivers' contributions.

## 4 Methods

### 4.1 Design

This study employed a qualitative descriptive design, which allows for a comprehensive summary of events in everyday language, staying close to participants' actual experiences and providing a straightforward yet rich depiction of the phenomenon under study (Sandelowski 2000). This approach is characterised by methodological flexibility, enabling researchers to apply both inductive and deductive analytical strategies as appropriate to the context and research aims, while remaining grounded in empirical data (Sandelowski 2000, 2010). Given the focus on shared experiences, dyadic data collection and dyadic data analysis were employed (Eisikovits and Koren 2010). The study was reported following the Consolidated Criteria for Reporting Qualitative studies (COREQ) checklist (see Supporting Information S1) (Tong et al. 2007).

### 4.2 Recruitment

The participants were recruited among those participating in a longitudinal study aimed at examining the self‐care of MCC older adult–caregiver dyads in Italy (De Maria et al. 2019). We used a purposive, maximum variation sampling strategy (Patton 2015) to recruit older adult–caregiver dyads representing the four dyadic care typologies described by Buck et al. (2013), diverse sex combinations (e.g., man older adult–woman caregiver, woman older adult–man caregiver), relationship types (e.g., spouses, adult children, others), and geographic areas (North, Central, and South Italy) (Buck et al. 2013).

### 4.3 Inclusion and Exclusion Criteria

The inclusion criteria for older adults were being aged 65 years or older and living with at least two chronic conditions. Caregivers had to be actively involved in managing the older person's conditions and be over 18 years old. Older adults diagnosed with dementia or cancer were excluded. Older adults with cancer were excluded, as cancer management often involves acute treatments (e.g., chemotherapy or radiotherapy) that require self‐care practices not typically associated with the management of chronic conditions. Individuals unable or unwilling to provide informed consent were also not eligible.

### 4.4 Data Collection

Data were collected between July and December 2024 through semi‐structured, in‐person interviews with older adults and their family caregivers. Each member of the dyad was interviewed separately, with the interviews conducted consecutively on the same day. This separate interview approach allowed each participant to express their perspective freely, without being influenced by the presence of the other dyad member, even though the other person was often referenced in the narrative. This method enabled a deeper understanding of how each individual experiences and interprets shared disease management. It also allowed for the comparison of two distinct perspectives during data analysis, highlighting both overlaps and contrasts, and supporting the construction of a dyadic perspective through researcher interpretation and synthesis (Eisikovits and Koren 2010).

Participants completed a socio‐demographic questionnaire collecting data on sex, age, education level, marital status, employment status, perceived income, cohabitation with caregivers, and types and number of older adults' chronic conditions. The dyads completed the Dyadic Symptoms Management Type (DSMT) Scale (Buck et al. 2013), a single‐item instrument that asks patients and caregivers to independently indicate who provides most of the care for the patient. Four response options are presented: the patient, the caregiver, or both members either collaboratively or in a complementary way. Based on their responses, dyads are classified as: patient‐oriented, caregiver‐oriented, collaborative, or incongruent when patient and caregiver responses are discordant (Buck et al. 2013).

Cognitive function was assessed using the Montreal Cognitive Assessment (MoCA) (Nasreddine et al. 2005). Scores range from 0 to 30, with a score of 26 or above generally considered within the normal range. Frailty was assessed using the Short Functional Geriatric Evaluation (SFGE) (Liotta et al. 2023), which consists of 13 items and allows classification of older adults into four categories based on total score: robust (≤ 0), pre‐frail (1–2), frail (3–9), and very frail (≥ 10).

An interview guide was developed by the research team and pilot tested in the first two interviews, leading to minor rephrasing (Kallio et al. 2016). Key topics included older adult self‐care and caregiver contributions to self‐care experiences, chronic disease prioritisation, and decision‐making processes within the dyads (Table 1). The interview questions were similar for both members of the dyad, with small adaptations to the respondent (e.g., for older adults: ‘Can you describe what you do on a daily basis to manage the chronic conditions you live with?’; and for caregivers: ‘Can you describe what you do on a daily basis to manage the chronic conditions of your family member?’).

**TABLE 1:** Interview guide.

Questions for older adult	Questions for family caregiver
Can you tell me which chronic conditions you live with?	Can you tell me which chronic conditions your family members live with?
Is there one condition, among those you mentioned, you consider the most important to monitor or manage more closely than the others?	Is there one condition, among those you mentioned, you consider the most important to monitor or manage more closely than the others?
If yes, why do you consider that condition more important?	If so, why do you consider that condition more important?
How do you make decisions about managing your chronic conditions?	How do you make decisions about managing the chronic conditions of your family member?
Have there been situations where you and your caregiver disagreed on how to manage your chronic conditions?	Have there been situations where you and your family member disagreed on how to manage his/her chronic conditions?
How do you handle disagreements when it comes to managing your chronic conditions?	How do you handle disagreements when it comes to managing chronic conditions of your family member?
Can you tell me what you do on a daily basis to manage the conditions you live with?	Can you tell me what you do on a daily basis to manage the chronic conditions of your family member?
Can you tell me what your caregiver does every day to help you manage your conditions	Can you tell me what your family member does every day to help you manage your conditions?
Managing multiple chronic conditions can be challenging. Could you tell me in which situations you have experienced more difficulty in managing your chronic conditions?	Managing multiple chronic conditions can be challenging. Could you tell me in which situations you have experienced more difficulty in managing the chronic conditions of your family member?
*Closing question*	
Is there anything else you would like to add to these topics, or anything you feel is important that we have not covered during the interview?	Is there anything else you would like to add to these topics, or anything you feel is important that we have not covered during the interview?

The data collection process consisted of the following phases.

1. *Pre‐interview*. Eligible dyads were contacted by telephone by three trained research assistants (GAB, IDI, VG), all female PhD students conducting this study as part of their doctoral research projects. They informed potential participants about the study's aim and, in case of consent, scheduled appointments for in‐person interviews at times and locations convenient for the participants. No dyads declined to participate.
2. *Qualitative interview*. The interviews were conducted separately with the older adult and the caregiver on the same day, with only the interviewer present. Both interviews were carried out by the same research assistants who had initially contacted the participants by telephone, to foster rapport and trust (DiCicco‐Bloom and Crabtree 2006). All interviews were audio‐recorded with participants' consent. Consistency in interview procedures among research assistants was ensured through a training course involving interview simulations, supplemented by continuous supervision and feedback from senior researchers. Recruitment continued until data saturation was reached, defined as the point at which no new information emerged from additional interviews (Kerr et al. 2010). The three research assistants each conducted approximately the same number of interviews.

### 4.5 Data Analysis

Descriptive statistics were used to describe the participants' characteristics, including mean, standard deviation (SD), median, interquartile range (IQR), frequencies, and percentages. All interviews were transcribed verbatim and anonymised prior to analysis. A hybrid content analysis approach was employed, combining both deductive and inductive strategies (Morgan and Hoffman 2018). The analysis began with a set of broad categories derived deductively from the interview guide, which provided an initial framework. This framework was then expanded and refined through an inductive reading of the data, allowing for the development of data‐driven codes, subcategories, and categories. Given that dyads were the unit of analysis, we employed a dyadic analysis method (Eisikovits and Koren 2010), which allowed for the examination of both individual and dyadic perspectives within each dyad. The dyadic analysis followed the following steps.

1. *Familiarisation with the data*. The interview transcript of one member of each dyad was read multiple times, accompanied by the writing of reflective notes to develop a deep understanding of the content.
2. *Initial coding at the individual level*. Meaningful units within the text were identified, and preliminary codes that captured the core ideas expressed were assigned. The same procedure was then applied to the interview transcript of the other dyad member.
3. *Dyadic‐level analysis*. The data were analysed at the dyadic level by examining areas of convergence and divergence between the two members' responses and codes. By comparing codes, subcategories were developed to highlight shared or contrasting perspectives and relational patterns (Eisikovits and Koren 2010). These subcategories were then grouped into categories.

To manage and organise the data, interview transcripts were imported into a Microsoft Excel spreadsheet. A comparative grid was created, with each row representing a dyad and each column a specific interview question. The transcripts of both dyad members for each question were placed side by side within each cell to enable direct comparison. Coding from transcripts and analytical notes was added in adjacent columns, along with emerging subcategories. This structure allowed for a dual‐layered analysis: vertically, comparing transcripts, codes, and subcategories across older adults and caregivers; and horizontally, examining transcripts within each dyad. Separate Excel sheets were created for each main category to facilitate a clearer and more accessible presentation of the findings. Table 2 provides an excerpt of the dyadic analysis process, while Table S1 in Supporting Information S1 presents the complete coding grid for one category.

**TABLE 2:** Example of dyadic analytic process for the category ‘management‐focused disease prioritisation in older adult–caregiver dyads’.

Dyad code/relationship	Participant responses (verbatim): most important condition to monitor/manage and reason^a^		Notes on disease priority comparison	Disease priority code		Notes on reason for disease priority comparison	Reason for disease priority code		Subcategory
	Older adult	Caregiver		Older adult	Caregiver		Older adult	Caregiver	
1 Father & daughter	‘[What matters to me is] this ache that comes at my hip’	‘Definitely the issue of blood sugar, diabetes’ ‘His blood pressure also tends to rise, and this is something that worries me too’	Father prioritises recent hip pain, while daughter focuses on diabetes and hypertension	Osteoarticular pain	Hypertension, diabetes	Father: the most recent health issue causing walking problems Daughter: diseases that are more concerning, and cause symptoms	Disabling symptoms	Higher risk of complications Most symptomatic disease	Disagreement on chronic disease considered a priority and reason
2 Mother & daughter	‘For me, hypertension is important because it caused my recent stroke. Yes, it directly triggered my stroke, high blood pressure along with cholesterol’	‘Blood hypertension, because for my mother it was a difficult experience. She had a TIA due to a hypertensive crisis, so she is very sensitive to this condition’	Mother & daughter agree on the importance of managing hypertension	Hypertension	Hypertension	Mother: disease that has previously caused stroke Daughter: disease that has previously caused TIA episode	Higher risk of complications	Higher risk of complications	Agreement on chronic disease considered a priority and reason
4 Husband & wife	‘Asthma causes me more problems when it happens [asthma attack] while I am working around people, and it's not pleasant’	‘Asthma, it triggers panic in him, and it takes a while to manage it, even after the attack passes, he remains very agitated. That's exactly why: because he gets scared, and it takes me time to calm him down and handle the situation’	Husband & wife agree on the importance of managing asthma	Asthma	Asthma	Husband: the social discomfort of managing asthma attacks in public Wife: breathing difficulties causes panic episodes	Perceived social impact	Perceived psychological impact	Agreement on chronic disease considered a priority and disagreement on reason

^a^ Translated quotes from Italian, preserving original phrasing.

Each interview was independently coded by two researchers: the research assistant who conducted the interview (GAB, IDI, or VG) and a senior researcher with expertise in qualitative research (MM). This dual‐coding approach ensured methodological rigour and enhanced reflexivity in the analysis. The coding outputs were then compared within the research team. Discrepancies were discussed and resolved through consensus.

### 4.6 Ethical Considerations

The current study was conducted in accordance with the ethical principles outlined in the Declaration of Helsinki (World Medical Association 2013). Approval from the Research Ethics Committee was obtained for the main study (Protocol number: ComET ASReM 2017/138). Informed consent was secured from all participants, with an emphasis on confidentiality and the voluntary nature of participation. Interviews were conducted in private settings to ensure confidentiality, and any identifying information was removed during transcription.

### 4.7 Rigour and Reflexivity

To ensure trustworthiness, the criteria of credibility, transferability, dependability, and confirmability were applied (Lincoln et al. 1985). Credibility was ensured through prolonged engagement with the data and independent coding. Confirmability and dependability were supported by maintaining a detailed audit trail throughout the research process, documenting methodological and analytical decisions. The process was supervised by a senior researcher with expertise in qualitative methods who was not directly involved in data collection. Transferability was addressed by providing rich descriptions of participants' socio‐demographic characteristics, the research context, and the analytical procedures, allowing readers to assess the relevance and applicability of the findings to other settings. Additionally, the use of dyadic analysis enabled the exploration of both individual experiences and relational dynamics, adding contextual depth and enhancing the applicability of results to similar situations. Member checking was not performed to avoid burdening participants and potentially compromising the authenticity of initial interview content through retrospective reinterpretation.

## 5 Findings

### 5.1 Characteristics of Participants

Thirty‐four dyads participated in the study. The older adults had a mean age of 80.09 years (SD 6.95), with equal representation of both sexes. Most had a low education level (≤ 8 years, 84.85%) and a median of 4.00 chronic diseases (IQR 2.75–5.00), with the most common being DM (73.53%), hypertension (58.82%), and HF (38.24%). The median MoCA score was 26 (IQR 20.50–28.00). In terms of frailty, 52.94% of older adults were robust, 23.53% were pre‐frail, 14.71% were frail, and 8.82% were very frail.

Caregivers were younger than older adults (mean 51.71 years, SD 14.59), predominantly female (82.35%), and had higher education levels (≥ 9 years, 79.41%). Most were the older adults' children (61.76%) and spouses (20.59%); 41.18% lived with the older adults. The most common gender configuration was woman older adult‐woman caregiver (44.11%) (Table 3). The mean duration of the in‐person interviews was 17 min.

**TABLE 3:** Socio‐demographic and clinical characteristics of older adults with multiple chronic conditions and their family caregivers (*n* = 34).

Variables	Patients (*N* = 34)	Caregivers			
		Overall (*N* = 34)	Children (*N* = 21)	Spouses (*N* = 7)	Other (*N* = 6)
Age (years), M (SD) *range*	80.09 (6.95)	51.71 (14.59)	50.38 (8.74)	69.43 (6.18)	35.67 (17.26)
	*66–95*	*18–78*	*33–65*	*59–78*	*18–60*
Sex, *N* (%)					
Female	18 (52.94)	28 (82.35)	17 (80.95)	6 (85.71)	5 (83.33)
Male	16 (47.06)	6 (17.65)	4 (19.05)	1 (14.29)	1 (16.67)
Education level (years), *N* (%)					
0–8	29 (85.29)	7 (20.59)	1 (4.76)	5 (71.42)	1 (16.67)
≥ 9	5 (14.71)	27 (79.41)	20 (95.24)	2 (28.58)	5 (83.33)
MoCA, median (IQR)	26 (20.50–28)				
Frailty index, *N* (%)					
≤ 0 (robust)	18 (52.94)				
1–2 (pre‐frail)	8 (23.53)				
3–9 (frail)	5 (14.71)				
≥ 10 (very frail)	3 (8.82)				
Marital status, *N* (%)					
Married	17 (50.00)	16 (47.06)	7 (33.33)	7 (100)	2 (33.33)
Unmarried	—	13 (38.24)	9 (42.86)	—	4 (66.67)
Divorced	1 (2.94)	4 (11.76)	4 (19.05)	—	—
Widowed	16 (47.06)	1 (2.94)	1 (4.76)	—	—
Employment status, *N* (%)					
Unemployed/retired	31 (91.18)	10 (29.40)	2 (9.52)	6 (85.71)	2 (33.33)
Employed	3 (8.82)	24 (70.60)	19 (90.48)	1 (14.29)	4 (66.67)
Perceived income, *N* (%)					
Less than needed	4 (11.76)	3 (8.82)	2 (9.52)	1 (14.29)	—
Enough for living	27 (79.41)	26 (76.47)	17 (80.95)	5 (71.42)	4 (66.67)
More than needed	3 (8.82)	5 (14.71)	2 (9.52)	1 (14.29)	2 (33.33)
Sex combination, *N* (%)					
Male_p_ + Male_c_	2 (5.89)		2 (9.52)	—	—
Male_p_ + Female_c_	13 (38.24)		7 (33.33)	6 (85.71)	—
Female_p_ + Male_c_	4 (11.76)		1 (4.76)	1 (14.29)	2 (33.33)
Female_p_ + Female_c_	15 (44.11)		11 (52.39)	—	4 (66.67)
Living with caregiver, *N* (%)					
Yes	14 (41.18)		7 (33.33)	7 (100)	—
No	20 (58.82)		14 (66.67)	—	6 (100)
Living area, *N* (%)					
Northern Italy	11 (32.35)	11 (32.35)	8 (38.09)	1 (14.29)	2 (33.33)
Central Italy	22 (64.71)	21 (61.77)	11 (52.39)	6 (85.71)	4 (66.67)
Southern Italy	1 (2.94)	2 (5.88)	2 (9.52)	—	—
N. chronic conditions, median (IQR)	4.00 (2.75–5.00)				
Chronic condition types, *N* (%)					
Diabetes mellitus	25 (73.53)				
Hypertension	20 (58.82)				
Heart failure	13 (38.24)				
COPD	8 (23.53)				
Hypercholesterolemia	7 (20.59)				
Chronic kidney disease	5 (14.71)				
DSMT scale, *N* (%)					
Patient‐oriented	7 (20.59)				
Caregiver‐oriented	4 (11.76)				
Collaborative	16 (47.06)				
Incongruent	7 (20.59)				

*Note:* Older adults' Frailty Index was measured by the Short Functional Geriatric Evaluation (SFGE) (Liotta et al. 2023).

Abbreviations: c, caregiver; COPD, chronic obstructive pulmonary disease; DSMT, dyadic symptom management type; IQE, interquartile range; M, mean; MoCA, Montreal Cognitive Assessment; N, sample number; p, patient; SD, standard deviation.

### 5.2 Qualitative Findings

Twenty‐five subcategories derived from 86 codes were grouped into five categories that described the older adult‐family caregiver dyadic experiences of managing MCCs (Table 4).

**TABLE 4:** Findings from dyads analysis.

Categories	Subcategories	Codes
Management‐focused disease prioritisation in older adult‐caregiver dyads	Agreement on chronic disease considered a priority and reason Agreement on chronic disease considered a priority and disagreement on reason	Disease with higher risk of complications Disease with disabling symptoms Disease with rapid onset of symptoms Most recent diagnosis Personal beliefs about disease Perceived psychological impact of the disease Perceived social impact of the disease
	Disagreement on chronic disease considered a priority and reason Disagreement on chronic disease considered a priority and agreement on reason	Disease with disabling symptoms Disease with rapid onset of symptoms Denial or unawareness of disease Disease with higher risk of complications Disease at high risk of decompensation Most recent diagnosis Most symptomatic disease Perception of disease severity Perception of uncontrollability of disease
Dynamics of decision‐making in disease management within dyads	Agreement on decision‐making responsibility: Older adult‐led decision making Caregiver‐led decision making Shared decision‐making	Older adult's desire for autonomy Older adult/caregiver medical expertise Family role Older adult/caregiver health condition
	Disagreement on decision‐making responsibility: Caregiver‐led vs. shared decision making Older adult‐led vs. caregiver‐led decision making Shared vs. caregiver‐led decision making Shared vs. older adult‐led decision making	Older adult's symbolic involvement in decisions Invisible support from caregiver Older adult/caregiver dominant role Older adult perceived autonomy
	Strategies for managing conflicts in decision making within dyads	Conflict and negotiation Conflict and imposition Conflict and resignation Discussion and delegation Discussion and persuasion Discussion and compromise Discussion and rational argument
Older adult's self‐care behaviours and caregiver contributions to self‐care	Collaborative care	Accompanying medical appointments Helping with medication management Going to pharmacy Giving advice Managing communication with physicians Support in personal hygiene Managing blood tests Managing relationships with healthcare services Medication management Diet management Scheduling medical appointments Preparing medications Managing prescriptions from GP Remembering medical appointments Remembering taking medications Supervising the diet Supervising medication intake Providing emotional support Providing instrumental support
	Older adult‐directed care	Medication management Diet management Blood glucose monitoring Body weight monitoring Blood pressure monitoring Engaging in physical activity Management of medical appointments
	Caregiver‐directed care	Preparing meals Assisting with personal hygiene Coordinating care with a care assistant Monitoring weight, glucose, and blood pressure Preventing respiratory crises Medication management Diet management Scheduling medical appointments Preparing medications Helping with medication management Managing prescriptions from GP
Challenges in managing multiple chronic conditions	Shared care challenges	Older adult's adherence to medications Older adult's adherence to prescribed diet Getting a diagnosis Acute event management Symptom management Accessing medical appointments Coordination among healthcare providers
	Older adult‐specific care challenges	Disease acceptance Performing physical activity
	Caregiver‐specific care challenge	Personal health issues Assisting with personal hygiene Acceptance of caregiving role
Roles in disease management and decision‐making	Single responsibility model Shared responsibility model Divided responsibility model: Caregiver‐led management with shared decision model Older adult‐led management with shared decision model Shared management and caregiver decision model Role discordance model	Autonomy in activities of daily living Older adult's cognitive status Older adult's age Functional impairment Character traits of older adult/caregiver Older adult‐caregiver cohabitation Quality of older adult‐caregiver relationship

Abbreviation: GP, general practitioner.

We reported verbatim quotes, each accompanied by a numerical code representing the dyad, participants' ages, familial relationship, and the older adult's MoCA and level of frailty to contextualise the data.

#### 5.2.1 Management‐Focused Disease Prioritisation in Older Adult‐Caregiver Dyads

This category describes how participants prioritised which chronic conditions to manage. By comparing the responses of both older adults and caregivers regarding the older person's chronic conditions with the medical records, we identified three patterns of disease awareness within the dyads: (1) both members reported all chronic diseases; (2) only one member did; and (3) neither the caregiver nor the older adult did. The most frequently omitted diseases were hypercholesterolemia, atrial fibrillation, and COPD.

We found that dyads could either agree or disagree on which chronic disease was most important to manage, as well as on the reasons behind their prioritisation (Table 4). Complete agreement occurred when both older adults and caregivers reported the same disease and the same rationale, often citing the risk of short‐ and long‐term complications or the rapid onset of symptoms.

Mother (2) *‘For me, hypertension is important because it caused my recent stroke. Yes, it directly triggered my stroke, high blood pressure along with cholesterol.’ (80 years, MoCA = 25, frail)*

Daughter (2) *‘Blood hypertension, because for my mother, it was a difficult experience. She had a TIA due to a hypertensive crisis, so she is very sensitive to this condition.’ (51 years)*

In some dyads, personal perspectives on the reasons for disease prioritisation differed. For example, one member might emphasise the social discomfort caused by symptom onset in public, while the other might focus on the psychological distress involved in symptom management.

Husband (4) *‘Asthma causes me more problems when it happens [asthma attack] while I am working around people, and it's not pleasant.’ (72 years, MoCA = 25, robust)*

Wife (4) *‘Asthma triggers panic in him, and it takes a while to manage it. Even after the attack passes, he remains very agitated. That's exactly why: because he gets scared, and it takes me time to calm him down and handle the situation.’ (65 years)*

Discrepancies emerged when older adults and caregivers identified different diseases as the most important to manage. Older adults tended to prioritise conditions that caused the most debilitating symptoms, limited mobility and autonomy, or provoked pain that worsened their quality of life. In contrast, caregivers focused on diseases that could lead—or had already led—to serious complications, such as hypoglycaemic coma or neuropathy in diabetes, or stroke in hypertension. These differing perspectives reflected a focus by older adults on their subjective experiences and the immediate impact of symptoms on daily life, and by caregivers on the prevention and management of disease‐related complications.

Father (1) *‘[What matter for me is] this ache that comes at my hip.’ (88 years, MoCA = 30, frail)*

Daughter (1) *‘Definitely, the issue of blood sugar, diabetes… Also, his blood pressure tends to rise, and that is something that worries me too.’ (59 years)*

In some cases, differences in identifying the most important disease stemmed from the older adult's lack of awareness or denial of having a chronic condition.

Mother (14) *‘Well, I have heart issues, high blood pressure, high blood sugar… The heart is important because if the heart stops, that's it’ (78 years, MoCA = 22, robust)*

Daughter (14) *‘Most important is COPD, because she struggles to walk and gets out of breath… She stops, then starts again. Right now, that's the disease causing the most symptoms and the greatest disability in her daily life.’ (41 years)*

In some cases, one member considered all chronic diseases equally important without prioritising a specific one, while the other identified a particular condition. These differences could arise from subjective perceptions of severity, possible underestimation of certain conditions, or personal health beliefs shaping the level of concern for specific diseases.

Father (10) *‘To be honest, I would keep all of them under control because they are all important.’ [Atrial fibrillation, DM, and HF] (83 years, MoCA = 26, robust)*

Daughter (10) *‘[It is important] atrial fibrillation… because it's the one that generally causes more problems if not treated. If it's a true atrial fibrillation and not temporary, due to heart failure, it could be fatal. And in the end, with atrial fibrillation, you have no choice but to call 112 [emergency service].’ (55 years)*

#### 5.2.2 Dynamics of Decision‐Making in Disease Management Within Dyads

This category describes recurring patterns in decision‐making about disease management, encompassing both agreement and disagreement over the primary decision‐maker (Table 4). Some dyads agreed that the older adult was the primary decision‐maker, although they might ask for advice from their caregivers or keep them informed of their decisions. The older adults' medical expertise and confidence in their own knowledge contributed to this pattern.

Mother (27) *‘First and foremost, I am a retired nurse, and I studied this field, so I know more than my daughter. But I always keep her informed about my decisions because she wants to stay updated.’ (74 years, MoCA = 27, pre‐frail)*

Daughter (27) *‘Well, the most important decisions are made by my mother. Of course, we always consult each other, I stay informed about medical appointments and any updates, and discuss everything together.’ (45 years)*

Some other dyads agreed that the caregiver was responsible for all health‐related decisions due to their medical expertise or the presence of a strong family bond and trust, particularly in parent–child relationships. Some dyads described a shared decision‐making process, characterised by open discussion and mutual trust, along with the caregiver's commitment to respecting the older adult's autonomy.

Mother (2) *‘When it comes to more complex decisions, I always ask for my daughter's opinion. For example, when considering a prosthesis [orthopaedic surgery]. I consulted her because I believe this was a crucial decision.’ (80 years, MoCA = 25, frail)*

Daughter (2) *‘Caring for someone is challenging, especially when it comes to defining boundaries. With my mother, I have always tried to maintain her autonomy in small and big decisions because preserving independence is important. But there are times when intervention is necessary, so I strive to mediate between respecting her autonomy and ensuring she takes care of herself.’ (51 years)*

We identified four types of discordance within dyads regarding who was in charge of decisions. In some cases, the older adult reported relying on the caregiver, while the caregiver described a shared decision‐making process.

Husband (26) *‘My wife is much better than me at making decisions… And when someone realises that the person they are with is more capable in some areas… then I am democratic enough to leave room for those who know how to do it better than me.’ (83 years, MoCA = 26, robust)*

Wife (26) *‘We decide together, we really do.’ (78 years)*

In other dyads, the older adults reported making decisions autonomously, while the caregivers emphasised their role in making decisions.

Father (13) *‘No one helps me [in making decisions]. I manage on my own. The physician makes decisions about my illness… my daughter has nothing to do with it.’ (80 years, MoCA = 22, robust)*

Daughter (13) *‘In theory, he decides but in practice, the whole family is involved. On paper, it's him, but, actually, it's us.’ (41 years)*

In some dyads, the older adult emphasised collaboration, whereas the caregiver attributed full responsibility to the older adult. Conversely, in other dyads, the older adult described a shared decision‐making process, while the caregiver viewed themselves as the primary decision‐maker.

Discrepancies in perceived decision‐making roles were shaped by the following factors: older adults' symbolic involvement in decisions, invisible support from caregivers, the dominant role assumed by either the older adult or the caregiver, and the older adult's perceived autonomy (Table 4). In some cases, caregivers made efforts to preserve the older adult's autonomy, identity, and authority, particularly when the older adult valued independence or had a background in leadership. However, decision‐making power remained with the caregiver, making the older adult's involvement more symbolic than substantive.

Father (30) *‘I've always done everything myself, you see. I was an army officer, so I took care of everything. Now, I need some help because I can't manage everything. But usually, I consult with them, my daughter, my son… I ask for advice, “What do you think? Should I do this or not?.”’ (85 years, MoCA = 28, robust)*

Daughter (30) *‘I make the decisions, since I am the eldest [child], and sometimes I have to assert myself with my siblings because we are three completely different people. But generally, we decide together. Dad has an important voice, of course, but he relies on me a lot; he has become very affectionate which he absolutely wasn't when he was young.’ (61 years)*

Some caregivers subtly influenced decisions, allowing older adults to feel they were making independent choices, even when the caregiver was guiding the decisions. In other cases, either the caregiver or the older adult assumed a dominant role in the decision‐making process, reflecting relationship dynamics or personality traits. Additionally, some older adults continued to view themselves as capable and self‐reliant despite functional or cognitive limitations; this perception did not always align with their actual abilities but reflected a long‐standing self‐image rooted in past roles and life experiences.

Dyads reported several strategies used in solving conflicts on decisions regarding disease management, which ranged from open discussion and compromise to imposition and passive acceptance. Some dyads agreed on adopting an approach based on dialogues, aiming to find solutions that could satisfy both parties. Other dyads reported combining open dialogue with persuasion, where the caregiver leveraged a sense of responsibility and emotional connection to influence their family member's decisions.

Grandmother (3) *‘I also say my opinion. But in the end, I say, “You [granddaughter] decide what's right.”’ (95 years, MoCA = 13, very frail)*

Granddaughter (3) *‘I try to explain what's best for her because she always tells me, “Don't let me die,” and I say, “If you don't want to die, you have to do what the physicians tell you and what we tell you. Otherwise, there's no point seeing all these physicians—we do this for you.”’ (32 years)*

In some dyads, discussions were unbalanced, with one member taking a more directive role in decision‐making as a protective effort to ensure timely and effective decisions for the older adult's good.

Father (37) *‘My son is very, well, very strict. I might be more like, “We can make an exception,” but in the end, I follow his lead, so I don't upset him’. (68 years, MoCA = 30, pre‐frail)*

Son (37) *‘We discuss things together. I just try to advise him in the best way possible. But let's say I try to convince him… I wouldn't say I have the final word, but I do try to be the one who asserts himself because I have more experience and can guide him better. But it's not that I want to override his will’. (33 years)*

In other dyads, decision‐making conflicts led to open arguments, which could lead to negotiation, imposition of one person's decision, or resignation by the other.

Father (6) *‘Maybe I can get a little mad, but these things pass’. (82 years, MoCA = 29, robust)*

Daughter (6) *‘He flares up a bit but once the agitation passes, we eventually find a solution calmly’. (46 years)*

#### 5.2.3 Older Adults' Self‐Care Behaviours and Caregiver Contributions to Self‐Care

This category describes the daily self‐care behaviours carried out by older adults, the support provided by caregivers in these practices, and the distribution of responsibility for managing chronic conditions within the dyad. We identified three types of disease care organisation within dyads: collaborative, older adult‐directed, and caregiver‐directed (Table 4).

In collaborative care dyads, older adults and their caregivers redistributed disease management tasks between them, with roles that could be either interchangeable or complementary. For example, caregivers might take charge of scheduling medical appointments, communicating with healthcare professionals, and managing administrative tasks, while their family members might focus on medication management, diet, physical activity, and monitoring blood glucose or blood pressure.

In older adult‐directed care dyads, older adults took full responsibility for all self‐care activities, including managing medications and diet, monitoring their physiological parameters, communicating with healthcare providers, and scheduling medical appointments. Caregivers were involved only minimally, offering advice and support when requested.

Mother (22) *‘I do everything on my own. I go to medical appointments, all the check‐ups, everything, I handle it all by myself.’ (84 years, MoCA = 14, robust)*

Daughter (22) *‘Well, mom is very independent, she knows how to manage all her treatments on her own. But when she needs help, either I or the other sister step in to assist her.’ (57 years)*

In caregiver‐directed care dyads, caregivers carried out most of the care tasks, such as handling administrative duties, communicating with healthcare providers, accompanying older adults to medical appointments, managing medication and diet, assisting with personal hygiene, and preparing meals.

Mother (16) *‘I don't do anything anymore… but I go to the bathroom on my own. I maintain a proper diet.’ (87 years, MoCA = 23, very frail)*

Daughter (16) *‘I prepare her medicine. I cook for her. I help her get into the shower. I always get everything ready for her, I have the pill organiser, so I manage that. I go to the GP, I take care of getting the prescriptions and if there's a medical appointment, I'm always the one who takes her.’ (62 years)*

In some cases, a home aide or care assistant was also involved, and the family caregiver had to coordinate the older adult's care with them.

#### 5.2.4 Challenges in Managing Multiple Chronic Conditions

This category describes the several challenges addressed by older adults and caregivers in managing MCCs. Some challenges were reported by both parties. For example, some dyads presented common difficulties in ensuring adherence to medications or dietary restrictions, particularly among those with diabetes and hypertension. Dietary challenges often stemmed from older adults' eating habits or their perception that dietary restrictions compromised the pleasure of food. Caregivers struggled to manage their family member's resistance to changing their habits.

Husband (29) *‘I wasn't a fan of sweets. But, since I found out I have diabetes, I've become addicted to sweets. If I'm at a restaurant and there's a cake, I already know I'll have it… Before, I didn't think about it at all. Yes, I used to eat them, but I wasn't obsessed with them.’ (76 years, MoCA = 24, robust)*

Wife (29) *‘Many times, he eats things he shouldn't, and I have to tell him ‘No’… I take the stuff away from him, saying ‘I'm doing this for your own good’. For example, if he sees a type of fruit that he can't eat, he'll take two, and I'll say, ‘Take it but maybe just one’.’ (74 years)*

One dyad reported difficulties in obtaining a timely and accurate diagnosis, while others reported difficulties in obtaining multiple prescriptions from their general practitioner (GP), booking and attending several medical appointments, or coordinating care among specialists.

Father (21) *‘The difficulty comes when I need a medical visit and have to book it. Then I always have to ask my daughter where to go, what to do, and to go to the GP to get the prescription. These things are sometimes a bit complicated for me.’ (81 years, MoCA = 28, pre‐frail)*

Daughter (21) *‘So, dealing with many illnesses is not easy because you have to coordinate everything with the GP, and with the various specialists. Making appointments, writing them down, remembering, accompanying him, and then all this in today's situation, with long waiting lists, is not exactly easy.’ (54 years)*

Some dyads struggled with managing symptoms such as coughing, asthma attacks, pain, incontinence, and nocturia, especially when occurring simultaneously. Others struggled with acute events, such as hypoglycaemia, which caused embarrassment and distress.

Husband (25) *‘I had fainting episodes due to metformin, which caused hypoglycemia. It happened two or three times, especially at work, and it was quite unpleasant. You know, people don't know, and they get worried when they see you faint in front of them. So, it was quite embarrassing.’ (66 years, MoCA = 29, robust)*

Wife (25) *‘Difficulties? When he fainted, one of the first times, because he was taking three metformin tablets a day, he fainted in the middle of a concert, and I was really scared… Well, those moments were full of stress, afterward I was upset for a couple of days.’ (59 years)*

Some challenges were reported only by older adults or family caregivers. For example, a son reported feeling embarrassed while assisting his mother with personal hygiene, while a daughter found it difficult to adjust to the caregiving role.

Son (7) *‘[After her hospitalisation], the issues were more practical. If my sister wasn't around, I was the one who had to wash her. So, there was my own embarrassment, and hers too. Her sense of modesty was being invaded, and I had to get used to it, because she wasn't able to do what a person normally does, from simply washing to getting dressed.’ (53 years)*

Daughter (15) *‘At the beginning, everything was difficult. Then, once you get into the routine [of taking care of her dependent mother], it doesn't really become easier, but you have to handle it.’ (45 years)*

A husband reported struggling to contribute to his wife's disease management due to his visual impairment.

Husband (24) *‘It's difficult for me to help her when she's not feeling well because of diabetes or cholesterol, since I'm visually impaired. I just tell her to take her pills.’ (68 years)*

Some older adults struggled to accept their chronic illnesses.

Mother‐in‐law (33) *‘When I got [the diagnosis of diabetes], I felt really bad. I was completely taken by surprise. At the time, I didn't even know about this disease. None of my parents or relatives had diabetes. No one, only me.’ (84 years, MoCA = 18, frail)*

#### 5.2.5 Roles in Disease Management and Decision‐Making

This category describes the role that older adults and caregivers play in the management of MCCs and in decision‐making. We compared the responses from dyads regarding the primary disease manager and decision‐maker. When older adults and caregivers agreed on their roles, we identified three patterns: one member was responsible for both disease management and decision‐making (i.e., caregiver or older adult); responsibilities were shared between older adults and caregivers; or different individuals took on the role of primary decision maker and disease manager.

When the older adult was responsible for both performing disease‐related care activities and making decisions, the caregiver contributed marginally by supervising, providing emotional support, or offering advice when requested. In other cases, the caregiver assumed both responsibilities, with the older adult being minimally involved, particularly in cases of cognitive impairment or functional dependence. Other dyads exhibited a collaborative approach, with older adults and caregivers handling the daily management of the older adult's chronic conditions and making health‐related decisions together. In some other cases, the older adults reported managing most aspects of their chronic conditions while relying on their caregiver for major disease‐related decisions.

Father (10) *‘I manage all by myself my illnesses daily, also because if I don't… I take my own medicines. I never forget my medicines.’ (83 years, MoCA = 26, robust)*

Son (10) *‘When it comes to my father, I am the one who makes the decisions… Daily, I do very little because my dad is highly independent. He manages his therapy perfectly, never forgetting a single dose. If he ever misses one, he immediately calls me to ask what might happen due to the missed dose.’ (55 years)*

Also, we identified dyads where the caregiver played the main role in disease management while the decision‐making was shared between the older adult and caregiver. Conversely, in other cases, the older adult managed the diseases independently while decisions were made collaboratively.

Husband (20) *‘I talk to her [when making decisions]. More than anything, she gives me advice. If she says “OK,” we do it, if she says “No,” we wait… In the morning, I measure my blood pressure, then I take 4 pills; after lunch I take cardioaspirin, and in the evening half a pill for cholesterol… Every year I do the electrocardiogram, then the heart ultrasound, and then the stress test. If you have treatment, you have to follow it.’ (71 years, MoCA = 27, robust)*

Wife (20) *‘We decide together, we say “Let's do this, let's do that.” No, it's not just him or me who decides. Besides, if I were the one deciding, he wouldn't listen to me. We have to do it together… It's easy for me to take care of him: just give him advice, make him eat well… He does everything by himself.’ (71 years)*

Finally, in some dyads, discrepancies were observed between older adults' and caregivers' accounts regarding their respective roles in disease management and decision‐making, suggesting divergent interpretations of their own responsibilities and potential role misalignment within the dyad. Several factors influenced the observed role patterns, including older adults' dependence in daily activities, cognitive and functional impairments, age, cohabitation with caregivers, the quality of the older adult‐caregiver relationship, and personal traits of the members of the dyad.

## 6 Discussion

Our study aimed to explore the experiences of managing MCCs within older adult‐caregiver dyads. To our knowledge, this is the first study to use a dyadic qualitative approach, which allowed us to capture the complexity of managing MCCs within dyads, providing a deeper understanding of their dynamics. We found that older adults and their family caregivers prioritise certain chronic conditions over others, adopt various strategies for decision‐making and conflict resolution, and face both shared and unique challenges in disease management. They also differ in how they distribute responsibilities and make decisions related to chronic conditions.

In our sample, family caregivers were predominantly women, reflecting the traditional gendered division of roles in Italian and Southern European cultures, where caregiving responsibilities for ill, younger, and older family members are typically assigned to women (Labbas and Stanfors 2023). Most caregivers were daughters, in line with norms of filial obligation, while a smaller number were wives—further reinforcing longstanding gender roles within marriage (Barigozzi et al. 2020). These cultural dynamics are essential to understanding how dyads experience and manage MCCs (Simmons et al. 2024). For example, older men may underreport symptoms or health problems to preserve their role as fathers or husbands, while daughters may overprotect their parents, taking on caregiving responsibilities even when not strictly necessary (Charenkova 2023). Due to our limited sample size, we were unable to examine the influence of kinship roles and gender on MCC management, an area that warrants further investigation through future quantitative research.

In our study, some dyads showed limited awareness of the older adult's chronic diseases. Several factors may explain this. Asymptomatic conditions, such as hypertension, hypercholesterolaemia, and osteoporosis, are often perceived as less significant or easily forgotten, while diseases with visible or disabling symptoms tend to be prioritised (Sathanapally et al. 2020). Furthermore, healthcare providers may inadequately communicate diagnoses; for instance, COPD is often labelled as bronchitis, obscuring its chronic, degenerative nature (Patel et al. 2024). Cognitive impairment in older adults can further hinder recall (Lovett et al. 2023). This can negatively affect self‐care, reduce caregiver support, and lead to poor disease management, treatment nonadherence, and delayed medical intervention, worsening health outcomes (Chimezie 2023; Magi et al. 2024). This finding highlights the need for nurses to strengthen health education and communication strategies that ensure both older adults and family caregivers possess accurate knowledge of chronic disease, supporting effective MCCs management.

Our findings also reveal variations in how older adults and caregivers prioritise chronic disease management. Older adults tend to prioritise conditions causing disabling symptoms, such as pain or mobility issues, while caregivers focus on diseases associated with serious complications (Riffin et al. 2018). These differences may reflect older adults' lack of knowledge, denial, or a lower level of education compared to caregivers (McGilton et al. 2018). When dyads agree on disease priorities, it may indicate shared understanding and effective communication (Ploeg et al. 2020). However, even when in agreement, their reasoning may differ; for example, older adults may focus on social consequences, while caregivers emphasise the psychological burden of managing symptoms. These differences highlight the diverse experiences shaping MCC management in dyads (Riffin et al. 2018). Nurses should recognise and integrate these different perspectives to ensure comprehensive care, facilitating conversations that bridge viewpoints and enhance disease awareness to improve health outcomes. Nurses can act as mediators within dyads, guiding structured discussions that align disease management and promote effective self‐care and caregiver support.

Our findings highlight the complexity of decision‐making when two individuals are involved. Dyads can agree on decision‐making roles, adopting shared, older adult‐, or caregiver‐led approaches. Shared decision‐making is characterised by open communication, mutual trust, and respect for the older person's autonomy, leading to greater satisfaction and improved treatment adherence (Pel‐Littel et al. 2023). Older adult‐led decision‐making can reflect personal expertise, a need for autonomy, or a desire for active involvement (Bujold et al. 2022). In contrast, caregiver‐led decision‐making can result from greater caregiver medical knowledge or the strong trust and reliance that older adults place in their caregivers. Discrepancies in perceived decision‐making roles are also common. For example, while older adults may acknowledge a decision‐making role for caregivers, caregivers may describe the dynamic as more collaborative. In such cases, caregivers may downplay their influence or intentionally adopt a supportive role to preserve the older adult's autonomy and, in the case of adult children, to respect familial roles and maintain the parent's dignity (Conway 2019; Luichies et al. 2021). Older adults may report autonomous decision‐making, while caregivers describe a more collaborative process. These discrepancies may reflect the invisible support caregivers provide to preserve older adults' dignity and traditional role, especially in dyads involving adult children (Conway 2019; Luichies et al. 2021; Riffin et al. 2018), or the older adults' enduring self‐perception as autonomous, shaped by past independence and not yet adjusted to the changes brought about by MCCs (Poitras et al. 2018). Our findings emphasise the importance for nurses to acknowledge the primary decision‐maker within dyads and to address role discrepancies through interventions aimed at improving communication and effective disease management. Furthermore, the findings highlight the role of nurses in facilitating shared decision‐making processes, ensuring that patients' autonomy is respected while also safeguarding patient safety.

We found that dyads adopt several conflict resolution strategies, ranging from collaborative approaches to more authoritative ones, in which either the caregiver or the older adult imposes a decision. These dynamics are shaped by the older adult's desire to maintain autonomy, the caregiver's sense of responsibility, the emotional bonds between them, and longstanding relational patterns. For example, caregivers may use persuasion as a way to balance respect for autonomy with their perceived duty to protect the older adult's well‐being (Russell et al. 2024). While such strategies may improve disease management, they also raise concerns about subtle forms of coercion that limit older adults' decision‐making independence (Bujold et al. 2022; International Alliance of Carer Organizations 2023). In some cases, especially when caregivers perceive older adults as vulnerable or at risk of making harmful choices, conflicts escalate into imposed decisions. Older adults, however, may still see themselves as capable decision‐makers, which can intensify tensions (Hem et al. 2023). These instances, especially within parent–adult child dyads, often reflect a shift in power and authority that marks a move toward paternalism (Lee et al. 2024). This shift can reconfigure the traditional generational hierarchy, with adult children assuming a dominant role in decision‐making. Rather than a temporary response, paternalistic dynamics may become embedded in the caregiving relationship, potentially diminishing the older adult's perceived agency and self‐worth (Von Humboldt et al. 2024). In contrast, collaborative approaches that involve open discussion and seek decisions that balance the older adult's values with caregiver concerns allow both parties to have a voice in decision‐making (Pel‐Littel et al. 2023). These findings highlight the importance of viewing decision‐making as a relational and dynamic process shaped by shifting power balances within dyads. Therefore, nurses should be trained in communication and conflict mediation skills that help them support families in navigating these power dynamics constructively. Future research should explore interventions that promote more balanced, collaborative approaches respecting both older adults' autonomy and caregiving responsibilities (Wagner et al. 2023).

Our findings revealed three distinct patterns of disease care organisation within dyads: collaborative, older adult‐directed, and caregiver‐directed, illustrating the variability in how responsibilities are distributed and negotiated within dyads. These patterns reflect a continuum from older adult autonomy to complete caregiver involvement, consistent with previously described dyadic management typologies in HF (Buck et al. 2019) and MCCs (De Maria, Erba, et al. 2023; De Maria, Lee, et al. 2023). Collaborative care dyads adopt a shared approach, with both parties assuming complementary or interchangeable roles. This model reflects an adaptive, balanced care organisation, promoting shared responsibility, mutual support, and reduced caregiver burden (Ferraris et al. 2024). In older adult‐directed care dyads, older adults assume full responsibility for their self‐care, with minimal caregiver involvement. Older adults can engage in adaptive strategies to maintain a sense of normalcy despite illnesses, allowing them to perform daily routines and maintain personal identity even when living with MCCs. This capacity for adaptation highlights the need to interpret self‐care behaviours and decision roles within the broader context of identity continuity (Andersen et al. 2020). In some cases, older adults may downplay their difficulties to appear self‐sufficient or to avoid worrying or burdening family members, assuming full responsibility for all self‐care tasks themselves (De Maria, Erba, et al. 2023; De Maria, Lee, et al. 2023). Caregiver‐directed dyads may be more common when older adults experience severe cognitive or functional impairments, suggesting that a decline in abilities may lead to a shift in responsibility from older adults to caregivers (Schulz et al. 2020). These findings highlight the importance for nurses of recognising dyadic care configurations to design tailored educational interventions that enhance empowerment and self‐care skills in older adult‐directed dyads or skill‐building and stress management in caregiver‐directed ones. Building on this assessment, nurses can then develop personalised care plans that adapt to each dyad's organisation of care, thereby improving treatment adherence and reducing caregiver strain.

We found that dyads face several challenges in managing MCCs, some of which are shared, while others are specific to either the older adults or the caregivers. A commonly reported difficulty for both parties is maintaining adherence to medication regimens and dietary restrictions. Older adults often struggle with lifestyle changes, particularly dietary modifications when food is one of a the few remaining sources of comfort and pleasure (Ferraris et al. 2024). For family caregivers, ensuring adherence to medications and diet can cause emotional strain when older adults resist these changes, leaving them feeling responsible for health outcomes (O'Conor et al. 2021). Both also reported difficulties in obtaining timely, accurate diagnoses, leading to frustration and a sense of abandonment while navigating a fragmented healthcare system (Kim et al. 2024). Managing multiple symptoms occurring simultaneously increases daily challenges (Baldan et al. 2024). Empowering dyads with knowledge and skills for symptom and crisis management could reduce adverse health outcomes and emotional distress (Riegel et al. 2022). Family caregivers face specific challenges, including emotional strain and embarrassment related to physical intimate care tasks, especially among male caregivers (Faronbi et al. 2019). Moreover, the caregiving role transition can be difficult, especially for those who are unprepared (Burgdorf et al. 2022) or who are managing their own health issues. For older adults, accepting chronic disease diagnoses can be emotionally challenging, with denial or resistance hindering treatment adherence and lifestyle changes (Fricchione 2023). Addressing these psychological barriers through counselling or peer support could help older adults better adjust and manage their conditions over time (Thompson et al. 2022). These findings call for nursing interventions that integrate psychological support, caregiver training, and system navigation assistance, highlighting the multifaceted role of nurses in chronic care management.

When comparing how older adults and family caregivers manage MCCs and make decisions, we found that responsibility for disease management and decision‐making can be assumed by one individual—either the older person or the caregiver—or shared between them. Interestingly, we found that some dyads divided these roles, with one member leading decision‐making and the other handling daily disease‐related tasks, or they shared responsibility for one aspect (i.e., disease management or decision‐making), while leaving the other responsibility to their counterpart. This finding offers new insights into the complexity of dyadic dynamics, as previous research has rarely examined the distinct roles dyadic members assume in the performance of self‐care behaviours and decision‐making. Previous studies suggested that self‐care ability necessarily implied decision‐making autonomy, or that impaired decision‐making limited engagement in self‐care practices (De Maria et al. 2021). Our findings reveal a more complex and variable distribution of roles within dyads, which may be attributable to changes in cognitive or functional abilities, which are more likely to occur in advanced age and in MCCs. In our study, due to the small sample size, we were unable to identify clear patterns linking the caregiver's relationship type with specific role distribution, for example, whether spouses were more likely to engage in collaborative decision‐making, or children in more caregiver‐oriented approaches. Future quantitative research could further explore these possible patterns. These findings highlight the need to assess the distribution of roles in decision‐making and performance of self‐care behaviours, ensuring that education is tailored to the appropriate individual. Nurses are in a unique position to perform such assessments and deliver differentiated education that supports both members of the dyads in complementary ways. For example, if older adults perform self‐care but caregivers are the primary decision‐makers, educational interventions should aim to enhance the self‐care behaviours older adults find most challenging, while for their caregivers, they should focus on strengthening decision‐making skills.

### 6.1 Limitations

Several limitations should be acknowledged. First, the sample consisted of older adults in a stable phase of chronic conditions; therefore, the findings may not be transferable to younger populations or those with more acute health trajectories. Second, characteristics of the Italian healthcare system may have influenced our results, as it is a publicly funded system that guarantees access to care for citizens with chronic diseases. Certain patterns of care organisation observed in this study may not reflect those in countries with different healthcare structures. Additionally, the familistic nature of Italian culture, where family members often play a central role in caregiving, may have shaped specific dyadic arrangements and dynamics that may not be transferable to other cultural contexts. Third, the same research assistant interviewed both members of the dyad consecutively, introducing a possible interviewer bias, as the interviewer might have been unconsciously influenced during the second interview by what was shared in the first interview. To reduce this bias, interviewers followed an interview guide and were trained to maintain a neutral attitude and were explicitly instructed not to disclose any information shared by one member of the dyad to the other. Fourth, interviews were conducted by three different research assistants. We recognise that different interviewers may elicit different responses from participants; however, this was mitigated through joint training and ongoing supervision to ensure consistency in data collection and analysis. Finally, the short duration of some interviews may have limited the depth of individual responses. This variation may reflect differences in communication style, emotional readiness, cognitive functioning, or participants' comfort with the interview process. Nonetheless, the use of a dyadic approach and cross‐case comparisons helped to ensure a rich and meaningful analysis.

## 7 Conclusion

This study explored the experiences of dyads in managing MCCs, highlighting the complex, relational, and dynamic nature of disease management within these partnerships. The management of MCCs emerged as a co‐constructed and evolving process, shaped by ongoing negotiations of responsibilities, priorities, and expectations between older adults and their caregivers. The identification of different dyadic care patterns illustrates the heterogeneity and fluidity of these dynamics, which may shift over time. The findings also reveal underlying tensions and asymmetries in how MCCs management is organised and experienced. Nurses are encouraged to consider these relational dynamics and support dyads through tailored, flexible, and dyad‐centred interventions that respond to both individual needs and the interdependent nature of caregiving relationships, acknowledging the evolving and sometimes conflicting nature of MCCs management.

## Author Contributions

All authors have agreed on the final version and meet at least one of the following criteria (recommended by the ICMJE): (1) substantial contributions to conception and design, acquisition of data, or analysis and interpretation of data; (2) drafting the article or revising it critically for important intellectual content. The authors affirm that the methods used in the data analyses are suitably applied to their data within their study design and context, and the statistical findings have been implemented and interpreted correctly. The authors agree to take responsibility for ensuring that the choice of statistical approach is appropriate and is conducted and interpreted correctly as a condition to submit to the Journal.

## Conflicts of Interest

The authors declare no conflicts of interest.

## Supporting information


**Data S1:** Consolidated criteria for reporting qualitative studies (COREQ): 32‐item checklist.

**Table S1:** Dyadic analysis of the category ‘management‐focused disease prioritisation in older adult‐caregiver dyads’.

## Data Availability

Data available on request from the authors. The data that support the findings of this study are available from the corresponding author upon reasonable request.
