# Supplementary material for: Older Adults' Self‐Care and Family Caregiver Contribution in Multiple Chronic Conditions: A Dyadic Qualitative Study
Source: J Adv Nurs. 2025 Oct 5;82(6):6444–63. doi: 10.1111/jan.70246 (PMC13176706; doi:10.1111/jan.70246)
Supplement: Supplementary file 2 — Table S1: Dyadic analysis of the category ‘management‐focused disease prioritisation in older adult‐caregiver dyads’. [file JAN-82-6444-s001.docx]

**Additional file 1**

**Table S1 Dyadic analysis of the category ‘Management-Focused Disease Prioritization in Older adult -Caregiver Dyads’**

| **Dyad**  **Code and roles** | **Participant responses (verbatim): most important condition to monitor/manage and reason** | | **Notes on disease**  **priority**  **comparison** | **Disease priority**  **Code** | | **Notes on reason for disease priority comparison** | **Reason for disease priority**  **Code** | | **Subcategory** |
| --- | --- | --- | --- | --- | --- | --- | --- | --- | --- |
|  |  |  |  | **Older adult** | **Caregiver** |  | **Older adult** | **Caregiver** |  |
|  | **Older adult** | **Caregiver** |  |  |  |  |  |  |  |
| 1  Father & daughter | "[important for me is] This ache that comes at my hip" | "Definitely the issue of blood sugar, diabetes."  "His blood pressure tends to rise, and this is something that worries me too." | Older adult prioritizes the recent hip pain, while caregiver believes that both diabetes and hypertension are important | Osteoarticular pain | Hypertension,  Diabetes | Older adult: the most recent health issue causing walking problems.  Caregiver: diseases that are more concerning, and cause symptoms | Disabling symptoms | Higher risk of complications  Most symptomatic disease | Disagreement on chronic disease considered a priority and reason |
| 2  Mother &  daughter | “For me, hypertension is important because it caused my recent stroke. Yes, because it directly triggered my stroke, high blood pressure along with cholesterol.” | “Blood hypertension, because for my mother it was a difficult experience. She had a TIA due to a hypertensive crisis, so she is very sensitive to this condition.” | Both agree on the importance of hypertension | Hypertension | Hypertension | Older adult: disease that has previously caused stroke.  Caregiver: disease that has previously caused TIA episode | Higher risk of complications | Higher risk of complications | Agreement on chronic disease considered a priority and reason |
| 3  Grandmother & granddaughter | “Actually, I don’t have any illnesses that I need to manage”.  ”No, I don’t remember anything. Ask my nice — she’s the one who take care of me.” | “The one that worries me most is kidney failure, because everything starts from there. So, it’s more about keeping her fluid intake and weight under control.” | Older adult is unable to answer the question while the caregiver identifies chronic kidney failure | None | Chronic kidney failure | Older adult: unable to answer the question (due to cognitive impairment?).  Caregiver: disease with a higher risk of complications, where controlling fluids and weight is crucial | - | Higher risk of complications | Disagreement on chronic disease considered a priority |
| 4  Husband & wife | “Asthma causes me more problems when it happens [asthma attack] while I am working around people, and it’s not pleasant.” | “Asthma, it triggers panic in him, and it takes a while to manage it, even after the attack passes, he remains very agitated. That’s exactly why: because he gets scared, and it takes me time to calm him down and handle the situation.”  “On the other hand, chronic bronchitis, when he gets a cough, fever, those things are easier to manage medically.” | Both agree on asthma | Asthma | Asthma | Older adult highlights the social discomfort of managing asthma attacks in public. He does not mention COPD.  Caregiver emphasizes the older adult panic episodes due to breathing difficulties. COPD exacerbations are manageable with medical treatment and do not have psychological repercussions | Perceived social impact | Perceived psychological impact | Agreement on chronic disease considered a priority and disagreement on reason |
| 5  Husband & wife | “I think hypertension, because it gives me, for example, palpitations. If I go uphill, I have to slow down.” | “Lastly no” | Older adult identifies hypertension while the caregiver does not mention any specific disease | Hypertension | None | Older adult: disease causing most symptoms.  Caregiver does not identify any disease and reson | Rapid onset of symptoms | - | Disagreement on chronic disease considered a priority |
| 6  Father & daughter | “Diabetes. Because he didn’t even want me to check myself every morning [referring to blood sugar monitoring], but I insisted - also because on the mornings when my blood sugar is a bit high, then I try to be more careful, you know.”  “The reason is because it causes damages.” | “Diabetes, absolutely. We’re scared - even if he hurts his foot, if he gets a wound, because sometimes he goes out, maybe does a bit of work, and it happens that he gets a cut, and it takes longer to heal. So we’re always very careful, especially with his limbs and feet, because we’re very afraid.” “And also the diet, because he would tend to eat.” | Both agree on diabetes | Diabetes | Diabetes | Older adult reports the disease that causes more damage.  Caregiver specifically mentions the risk of diabetic foot complications and the need for a specific diet. | Higher risk of complications | Higher risk of complications | Agreement on chronic disease considered a priority and reason |
| 7  Mother & son | “Well, surely the heart. I think so, and in my ignorance, I believe it’s what controls us. I could be wrong. Because it’s life. In Naples [Italian city] they used to say: ‘the spleen, the heart, and the liver are what we all have and what give us life’. Neapolitans aren’t wrong. They make noise, do silly things, because not everyone went to school. But I’ll tell you, those old ladies, when they told you something, for me it was beautiful” | “The heart, because it was the most serious issue. Also, because she was dead in the kitchen, and then when they reached her chest muscle, breaking her ribs, they still managed to bring her from zero to ten. So, the fear was about that.” [referring to an episode of cardiac arrest] | Both agree on the “heart disease” but for different reasons | Heart disease | Heart disease | Older adult: according to popular belief, heart is one of the main vital organs.  Caregiver: because the older adult had a cardiac arrest as a consequence of the disease | Personal beliefs | Higher risk of complications | Agreement on chronic disease considered a priority and disagreement on reason |
| 8  Father & son | “It would be better not to have any illnesses and not have to take medications and not be 80 years old. Because when you’re over 80, a lot of things happen.... Neither hypertension nor diabetes worry me.” | “No, no particular problems” | They do not identify a priority disease | None (old age) | None | Older adult does not identify a disease but he considers old age to be the main issue.  Caregiver: no disease identified | Personal beliefs | - | Agreement on chronic disease considered a priority (no priority) |
| 9  Mother & daughter | “Osteoporosis because of the bones. I’ve had a total of 16 surgeries. I had hip replacement surgery twice - the first time in '94, then again in 2019. I also had it on my left hip and both knees. I had shoulder surgery, both under and over. These are all the surgeries I’ve had.” | “Well, what scares me is diabetes.” “Because when she was decompensated, we found out that she was probably forgetting to take her insulin... she had blood sugar spikes of 350, even 400, and at that point I didn’t see any way out.” | Older adult identifies bone-related issues, specifically osteoporosis while caregiver considers diabetes | Osteoporosis, Osteoarthritis | Diabetes | Older adult indicates osteoarticular problems that led to multiple surgeries.  Caregiver identifies diabetes, which previously became decompensated, leading to a need for more careful monitoring | Higher risk of complications | High risk of decompensation | Disagreement on chronic disease considered a priority and reason |
| 10  Father & daughter | “No, honestly, I would keep all of them under control because they’re all important.” | “Yes, the atrial fibrillation, so the whole heart-related aspect is what worries me a bit.”  “Yes, because more or less that’s the one that can cause bigger problems if you don’t intervene, especially if it’s real fibrillation and not just a temporary, passing episode caused by mild decompensation. That’s definitely the one that worries me the most, because it could be serious, and it’s also harder to manage if you’re not at home… with atrial fibrillation you have to intervene by calling 112 [emergency services].” | Older adult considers all illnesses equally important, while caregiver identifies cardiac diseases, especially atrial fibrillation. | All | Atrial fibrillation | Older adult: all diseases are equally important.  Caregiver: atrial fibrillation cannot be managed at home as require urgent medical treatment | - | Higher risk of complications | Disagreement on chronic disease considered a priority |
| 11  Mother & daughter | “Diabetes… because if I eat less, my blood sugar doesn’t go up.” | “Surely blood sugar [diabetes] is the most important, even though the issue of high blood pressure isn’t something to ignore either… The only thing that really scared me was the stroke he had. It was because they had taken away all his medications. I didn’t like that decision. In fact, after they stopped the meds, a stroke happened a few days later. Because his cholesterol was high.” | Both recognize diabetes as most important. Caregiver adds hypertension | Diabetes | Diabetes,  Hypertension | Older adult: disease with immediate effects if untreated.  Caregiver highlights diabetes and hypertension, the latter having led to a stroke | Rapid onset of symptoms | Higher risk of complications | Agreement on one chronic disease considered a priority and disagreement on reason |
| 13  Father & daughter | “Because… well, my wife lost a sister to this disease [diabetes], and there was a nephew, about fifty years old, and he too passed away because of diabetes. So, you get scared.”  “Because diabetes might cause me trouble, while as for breathing, I’m fine, no problem there.”  “They put a little machine on me, but it was useless [CPAP].” | “I think COPD, because it’s not well managed.”  “The diabetes overall he keeps it under control… As of today, the other one [COPD] is more important because you can see it [COPD symptoms].”  “The thing is, he denies a lot of things, so for example if we go out, we often have to stop for a bit and let him catch his breath.” | Older adult recognizes diabetes and denies having COPD, while caregiver indicates COPD | Diabetes | COPD | Older adult: disease that caused deaths in the family due to complications; he does not acknowledge COPD.  Caregiver: COPD is not managed by older adult and is symptomatic, while diabetes is under control | Denial or unawareness about disease  Higher risk of complications | Disabling symptoms | Disagreement on chronic disease considered a priority and reason |
| 14  Mother & daughter | "Well, the heart, in my opinion, is the most important one, but honestly, I think they’re all important because if you don’t keep diabetes under control, you’ll have heart problems too… And the heart is important, because if your heart stops, that’s it. So, I wouldn’t know which one is more important."  "If in the moment my heart, blood pressure, or diabetes is acting up, I worry more about that one [hypertension]. But it’s not like I neglect the others, you know." | “[most important] COPD, because she struggles to walk and gets short of breath. And in the morning especially, it’s a whole thing — she walks slowly, stops, then starts again."  "Because right now it’s the one causing the most symptoms and is the most disabling in her daily life." | For the older adult, heart disease along with diabetes and hypertension.  For caregiver is COPD | Heart disease, Diabetes,  Hypertension | COPD | Older adult: heart disease is tied to life itself, and diabetes and hypertension can affect the heart too.  Caregiver identifies COPD as the most disabling condition because its symptoms, especially breathlessness, have the biggest impact on the older adult’s everyday life | Denial or unawareness about disease  Higher risk of complications | Disabling symptoms | Disagreement on chronic disease considered a priority and reason |
| 15  Mother & daughter | "I see them all the same, because I have treatments for all of them."  "By now, I’ve gotten used to it." | "Kidney failure. If it hadn’t reached certain levels, we wouldn’t be in this situation now. It got to the point where she couldn’t breathe anymore. Heart failure happened as a consequence. That’s when the nephrologist got involved. From the moment we relied on her for kidney failure, everything else started to improve."  "It all started with kidney failure, it was a chain reaction, a domino effect." | Older adult does not identify any specific disease, while caregiver identifies kidney failure | All | Congestive kidney failure | Older adult does not identify any disease Caregiver identifies kidney failure as it triggered a cascade of other health problems | - | Higher risk of complications | Disagreement on chronic disease considered a priority |
| 16  Mother & daughter | "My back, because I couldn’t even lie in bed." | "Well, let’s say hypertension is the disease that worries me the most… I’m always afraid because of the dizziness she’s had, that it could lead to a stroke or something like that." | Older adult prioritizes back pain while caregiver hypertension | Osteoarticular pain | Hypertension | Older adult: prioritizes back pain, as it causes significant discomfort and limits mobility.  Caregiver: fears the risk of a cerebrovascular event, given the older adult’s family history (her father died of a stroke) | Disabling symptoms | Higher risk of complications | Disagreement on chronic disease considered a priority and reason |
| 17  Mother & daughter | "Diabetes, because if I get anxious, my blood sugar shoots up… so I try to eat less." | "Well, they’re all a bit connected, but right now it’s this neuropathy — because I really hope it gets better, and that next summer we won’t have to deal with all these symptoms again." | Older adult identifies diabetes while caregiver the diabetic neuropathy | Diabetes | Diabetic neuropathy | Older adult: diabetes quickly triggers symptoms and blood sugar increases in stressful situations.  Caregiver: diabetic neuropathy is the most recent and has caused troublesome symptoms | Rapid onset of symptoms | Perception of severity  Most recent  disease | Disagreement on chronic disease considered a priority and reason |
| 18  Grandmother & granddaughter | "Diabetes. Because I don't know what to eat… sometimes my blood sugar’s high, sometimes it drops."  "I take my meds, but it feels like sometimes they don’t work. It worries me because you can feel diabetes when it’s low, and you feel it when it’s high too. I sense it inside me before even measuring it."  "Yeah, diabetes is the one I've paid the most attention to, out of everything." | "Diabetes, because not following the right diet causes her blood sugar to swing, which could lead to other problems." | Both older adults and caregiver identify diabetes | Diabetes | Diabetes | Older adult: diabetes causes immediate symptoms if not treated.  Caregiver: diabetes causes symptoms if not treated, with possible consequences. | Rapid onset of symptoms | Rapid onset of symptoms  Higher risk of complications | Agreement on chronic disease considered a priority and reason |
| 20  Husband & wife | "The pump — the heart — because I think it’s the most dangerous. The heart doesn’t give you a warning. When I had my heart attack, I got up in the middle of the night with this sharp pain here, like stabbing knives. Then I was sweating… I’d make myself some chamomile tea and it seemed ease off." | "I think the blood pressure, because if it’s high you need to get it checked."  "One morning, while I was getting my infusion therapy at the outolder adult clinic, the doctor comes in and says, ‘Hey, your husband’s not feeling well.’ I asked, ‘Why? What happened?’ and she says, ‘His blood pressure’s 240.’ They kept him under observation. I remember that moment so clearly. I was really scared that time." | Older adult identifies the heart disease while caregiver hypertension | Heart disease | Hypertension | Older adult prioritizes heart disease due to the risk of sudden cardiac events, as experienced in the past (heart attack).  Caregiver focuses on hypertension because of a hypertensive crisis the older adult previously had | Higher risk of complications | Rapid onset of symptoms | Disagreement on chronic disease considered a priority and reason |
| 21  Father & daughter | "High blood pressure. Because I have to keep checking it, and I’m scared… that it might get too high and cause problems." | "In my opinion, hypertension is the most important, because if there are sudden spikes, he might get headaches or be unable to manage a hypertensive crisis. So, I consider it the most concerning." | Both older adult and caregiver identify hypertension | Hypertension | Hypertension | Older adult worries about possible problems if blood pressure gets too high.  Caregiver is concerned about hypertensive crises and their consequences | Higher risk of complications | Higher risk of complications | Agreement on chronic disease considered a priority and reason |
| 22  Mother & daughter | "My eyes." "Because I have glaucoma. And my mother was blind. She lost her sight very, very young." | "The way I see it, probably the glaucoma. Because she has diabetes too, but she’s older now and she manages it really well, she eats right, doesn’t make mistakes. The glaucoma, even though she’s had it for years and it’s under control, might be more problematic in the future. For the consequences it could bring…." | Both older adult and caregiver identify glaucoma | Glaucoma | Glaucoma | Older adult associates her glaucoma with her family history, as her mother went blind.  Caregiver expresses concern regarding possible future complications | Higher risk of complications | Higher risk of complications | Agreement on chronic disease considered a priority and reason |
| 23  Granmother & grandson | "No, for me they’re all important." | "Maybe the heart, because diabetes is there, sure, there are slip-ups, but it’s not excessive, it’s manageable. The fatigue though, the shortness of breath now and then, that’s what I think is more important. The effort she can’t make anymore, that matters more." | Older adult: considers all conditions equally important while caregiver sees heart disease as the most significant | All | Heart disease | Older adult considers all conditions equally important.  Caregiver sees heart disease as important due to symptoms like breathlessness and reduced physical stamina | - | Most symptomatic disease | Disagreement on chronic disease considered a priority |
| 24  Wife & husband | "To me, cholesterol seems more important, maybe because they tell me if it goes up, you can get plaques, and then you might need to have stents put in. I guess it’s the illness that scares me a bit more than the others. In fact, we don’t really eat fried foods, I mean my husband and I… And yeah, I’ve got the start of some plaques in my carotids. The diabetologist said, ‘Well, we’ll check it again in a year,’ but it’s nothing serious. The ophthalmologist said my cataract’s also starting to get a bit worse. I don’t know, cholesterol scares me more than diabetes itself. With diabetes, I feel like if I eat less, avoid sweets, give myself the shot, I manage it better." | "Diabetes. Because it affects the whole body. I see her when she’s down or not feeling well. She doesn’t have the energy to do things around the house, so I try to help her out." | Older adult identifies hypercholesterolemia while caregiver diabetes | Hypercholesterolemia | Diabetes | Older adult prioritizes high cholesterol due to the risk of artery blockages and complications, while diabetes is more manageable through diet and medicines.  Caregiver considers diabetes important due to complications on multiple organs and how its symptoms affect the older adult’s daily life | Higher risk of complications | Higher risk of complications  Disabling symptoms | Disagreement on chronic disease considered a priority and agreement on reason |
| 25  Husband & wife | "Diabetes worry me because of the long-term impact, if it’s not kept under control, it can cause all sorts of problems over time, gradually affecting different organs. It can make old age really disabling. But honestly, what bothers me more right now is hip osteoarthritis, it limits my movement and getting around, and it’s more of a short-term hassle. In the very short term, it’s osteoarthritis; in the long term, it’s diabetes. I mean, I’d like to reach an older age in good health, that’s kind of my goal, so I have to keep an eye on both. But while osteoarthritis is frustrating now because it stops me from doing physical activity, and I’ve always been pretty sporty, not professionally, but quite active, it’s the diabetes that I see as a long-term issue, something that could really disable me down the road." | "Diabetes, because as far as I know, it affects the whole body and can cause problems in lots of different areas. Definitely diabetes. Then there’s also his osteoarthritis, like when he drops something on the floor, you can see he can’t bend down naturally to pick it up. Or putting on a sock, he has to make these awkward moves to get it on. But [diabetes] is something that’s there and it’s going to stay. Whereas with the surgeries, he managed to fix one hip, and when he has the other surgery, that issue will be sorted out too." | Both older adult and caregiver identify diabetes and coxarthrosis | Diabetes, Coxarthrosis | Diabetes, Coxarthrosis | Both acknowledge that diabetes causes long-term complications and affects multiple organs.  Older adult reports that hip osteoarthritis leads to disabling limitations that reduce his mobility.  Caregiver recognizes the mobility difficulty caused by osteoarthritis | Higher risk of complications  Disabling symptoms | Higher risk of complications  Disabling symptoms | Agreement on chronic disease considered a priority and reason |
| 26  Husband & wife | "Well, diabetes. I manage the rest. Just yesterday I went for an ultrasound of the supra-aortic vessels, and I also did one on my legs because the diabetes doctor asked me to."  "It’s because I can’t control [the diabetes]. The other things I keep under control. I take the medications I’m supposed to. For example, my triglycerides are fine now because I took the meds. I do what they tell me for everything else, but with diabetes, I just can’t manage to do what they tell me." | "For me, it’s the kidneys. That fear they put in me about the kidneys nothing else ever did that. I know what he needs to eat for his diabetes, and if I manage it well, his blood sugar doesn't go up too much… Because kidneys are sneaky. The kidneys scare me, maybe because I saw him in that condition [referring to a kidney failure episode]." | Older adult identifies diabetes while caregiver chronic kidney failure | Diabetes | Chronic kidney failure | Older adult considers diabetes important because he struggles to keep it under control.  Caregiver considers kidney disease because its symptoms are subtler, and it has previously put the older adult’s life at risk | Perception of uncontrollability | Higher risk of complications | Disagreement on chronic disease considered a priority and reason |
| 27  Mother & daughter | "Well, it’s hard for me to answer this question because both diabetes and hypertension are important, obviously. Hypertension, as we know, raises blood pressure and it’s like you have this strong current of blood hitting your organs, even the brain. And since I used to work in neurosurgery, I know hypertension is the nasty one that can lead to a stroke. With diabetes, you kind of notice it, you might feel a little off, a bit dazed, or really thirsty even if it’s not a hot day. Then you check your blood sugar with a test strip and you see right away if it’s high. You should know I don’t eat a lot of sweets… I watch my diet carefully. And to tell you the truth, since I’ve been on this new medication, I don’t get that crazy hunger anymore…”  “So honestly, I’m less scared of diabetes than I am of the hypertension. Especially because in the past I had a hypertensive crisis, and they had to adjust my medication. I used to take one type of pill, now I’m on a different one that also has a bit of diuretic in it, and, in the evening, I take half a pill too. Now I never go over 140. But yeah, the blood pressure scares me." | "At the moment I’d say probably hypertension too. As for diabetes, mom’s started a new treatment, she’s on a new medication now and it’s under control, so it doesn’t worry me too much. I’d say hypertension, because of those sudden spikes she’s had before that’s what we have to keep an eye on now." | Both older adult and caregiver identify hypertensin | Hypertension | Hypertension | Older adult: because of the consequences it can cause.  Caregiver: because of the blood pressure spikes the older adult has experienced in the past | Higher risk of complications | Higher risk of complications | Agreement on chronic disease considered a priority and reason |
| 29  Husband & wife | "Diabetes is the worst thing I have. It makes damages."  "The time I really felt like a brick fell on my head was when they told me I had diabetes. I used to live eating and drinking out at restaurants every day… and then diabetes hits you… what do you do? I was a big eater, and now I’ve lost 17 kilos too…" | "Diabetes. Because so many times he eats things he shouldn’t, and I have to step in and say ‘no.’ I take the food away, ‘I’ll make you this instead, or that.’ For example, if he sees a kind of fruit he’s not supposed to eat, he might grab two, and I’ll say ‘no, maybe just one.’ He loves sweets too, and I have to tell him ‘no!’ So I have to watch out."  "Because the most important thing for him is really diabetes. The back pain can be treated, for the heart there’s a pill and when we see the doctor it’s fine, but diabetes is a disease that, even if you don’t notice it, you have to keep it under control, even more than the heart. Because with the heart, if you get chest pain you go to the doctor, or you have a heart attack and that’s it. But diabetes is a sneaky disease. Slowly, slowly…."  "With diabetes you can get kidney failure. The kidneys stop working, and then you need dialysis. And your feet, if you get a wound in your foot, it can turn into gangrene. I hear about so many people who have to be really careful with foot hygiene and all that." | Both older adult and caregiver identify diabetes | Diabetes | Diabetes | Older adult: it can cause damage.  Caregiver: it is a silent disease that leads to long-term complications affecting the kidneys, circulation, and risk of infections | Higher risk of complications | Higher risk of complications | Agreement on chronic disease considered a priority and reason |
| 30  Father & daughter | "The heart. I had heart surgery — a valve wasn’t working, and they replaced it, but it left me, how should I say, with a certain cardiac weakness. Maybe also because of the kind of work I used to do — a very, very difficult job, let’s be honest, I was a spy. I worked abroad, dealing with strange people. So I always had to stay alert, constantly looking around, watching who you speak with and knowing what you say, it’s a constant tension that gradually wears you down."  "The heart, in particular, worries me because it’s the most delicate thing, let’s say. I have to be careful with it. I can’t live carelessly, you know? Whatever I do, eat, drink, sleep, go for a walk, watch something, I always have to think, will this harm my heart, or something like that, you know what I mean? The constant worry of the possible consequences." | "In my opinion, the heart, because whatever other illness my dad might catch, even a simple flu or something like that, I worry because his heart isn’t strong, you know? He always has to be careful not to catch a cold, yes, more than diabetes, because now with diet and medications he manages that quite well. It’s mostly this, because in the end, I think the heart is the engine of everything. If a person has a strong heart..."  "Because he had to have several stents placed, and anyway his circulation isn’t good. And even when he had pneumonia last year, it was a bit serious because of his heart condition [heart failure]. We were supposed to have a coronary angiography done to see if more stents were needed, but we three children decided it was too invasive for the benefit it could bring, considering his age and after consulting several cardiologists, so we ruled it out." | Both older adult and caregiver identify heart disease | Heart disease | Heart disease | Older adult considers heart disease due to the possible consequences.  Caregiver: heart disease because of complications it has already caused (cardiac stents) and the potential future complications | Higher risk of complications | Higher risk of complications | Agreement on chronic disease considered a priority and reason |
| 31  Mother & daughter | "My leg and my back are always hurting."  "Let’s say my stomach and intestines worry me a little, 'cause you know, sometimes you feel bad when you go to the bathroom, here and there, you get it? And then I’ve got stomach issues too, in fact, I go for a gastroscopy every year with the gastroenterologist, so yeah…” | "Definitely the lung situation, that's what we’re always really, really careful about, because for her, a simple cold turns into bronchitis at best. So basically, she lives in a little protective bubble to avoid catching the flu or anything, because if she does, it’s a big problem." | Older adult identifies back pain and gastrointestinal problems while caregiver considers pulmonary disease | Musculoskeletal and gastrointestinal problems | COPD | Older adult points out back and leg pain, and gastrointestinal problems because of the symptoms they cause.  Caregiver highlights pulmonary disease because of the serious complications that respiratory infections could trigger given the older adult's vulnerability | Disabling symptoms  Denial or unawareness about disease | Higher risk of complications | Disagreement on chronic disease considered a priority and reason |
| 32  Mother-in-law & daughter-in-law | "This heart failure came out just now, with the last check-up I did. The physician who saw me this time found this heart failure." | "I’d definitely say heart failure, because since she already had a heart attack in the past, I’m always a bit worried it could happen again. And then there’s also chronic respiratory failure. Honestly, she has very little breathing capacity, so those are the two things that seem most critical to me." | Both older adult and caregiver identify heart failure; caregiver adds chronic respiratory failure | Heart failure | Heart failure,  COPD | Older adult points to the most recently diagnosed condition (heart failure).  Caregiver identifies heart failure, considering the previous heart attack, but also sees chronic respiratory failure as important due to the breathing difficulties affecting the older adult’s daily life | Most recent diagnosis | Higher risk of complications  Disabling symptoms | Agreement on chronic disease considered a priority and disagreement on reason |
| 33  Mother-in-law & daughter-in-law | "All illnesses [are important]. Every little thing is a problem, and you never really know which one is going to turn out worse." | "Diabetes — but not so much for keeping the blood sugar under control, it’s more for what comes with it. Like taking her twice a week to get her dressing done, taking her to the hospital where the Diabetic Foot Center is. And then when you see that bit by bit they’re taking away a piece of a toe, that really breaks your heart because you don’t know how to help her. Even just not being able to wear the shoes she used to, she’s been stuck with diabetic shoes for three years now… it’s tough." | Both older adult and caregiver identify diabetes | All | Diabetes | Older adult considers all illnesses important  Caregiver focuses on diabetes, emphasizing its physical and emotional consequences, like foot ulcers, amputations, and the loss of normal routines and independence | Perception of uncontrollability | Higher risk of complications | Disagreement on chronic disease considered a priority and reason |
| 35  Aunt & nice | "Diabetes. I’ve had hypoglycemia twice, ended up in the hospital… I had, what’s it called, a syncope. My leg was hurting, I took X [ketoprofen], didn’t know it would trigger diarrhea. I felt sick at night. But I don’t remember anything ‘cause they found me on the floor in the morning… Luckily my son-in-law came by, he was dropping off the dog before going to work. He found me unconscious on the floor. They gave me sugar and I came around. So, I think it was hypoglycemia. That’s what scares me. Every night I check my sugar levels to see where I’m at…”  "Because if I check and it’s low, I eat something extra, you know? So I don’t run into problems. ‘Cause I’m the kind of person who sleeps, I go to sleep at midnight and get up in the morning… So, if something happens, I stay there ‘cause I’m alone." | "Diabetes. Because she loves cooking, and there could be times when she eats more than she should without realizing it. Or since she lives alone, if she has a hypo or hyper at night, we wouldn’t know about it." | Both older adult and caregiver identify diabetes | Diabetes | Diabetes | Older adult highlights the fear of hypoglycemic episodes that have already endangered her life, especially because living alone makes nighttime episodes risky.  Caregiver worries about both hypo- and hyperglycemia, especially since the older adult lives alone and such episodes might go unnoticed | Rapid onset of symptoms  Higher risk of complications | Rapid onset of symptoms | Agreement on chronic disease considered a priority and reason |
| 36  Father & daughter | "Heart disease, I had those bypass surgeries."  "Because [when I had the surgery] the anesthesiologist said ‘It’s do or die,’ because if I didn’t get operated on, I wouldn’t be here now." | "Heart failure. Because if that [the heart] stops working, nothing else works."  "I’ve always paid more attention to heart failure than anything else. Not that the other issues don’t have meds or treatments, but clearly — like I always say — when the heart doesn’t work, everything else falls apart too. So we focus on that first, to keep everything else in check." | Both older adult and caregiver identify heart failure | Heart failure | Heart failure | Older adult highlights the seriousness of heart disease, recalling it led to major surgery that could have been fatal.  Caregiver considers heart failure the priority because the heart is the body’s most vital organ, and if it fails, it triggers a cascade of other complications | Higher risk of complications | Higher risk of complications | Agreement on chronic disease considered a priority and reason |
| 37  Father & son | "Definitely diabetes. Because my father had diabetes and so did my grandmother, so there’s a hereditary factor, and they both had complications in their lives." | "Actually, both are important, but for his quality of life, I’d say diabetes stands out more in his case. He’s also had surgeries on his lower limbs because of vein issues. So, the chronic complications, both in the small and large blood vessels, are significant, especially because they could limit his independence. And considering he’s still active, moving around, doing things on his own, I think it’s even more important to keep a close eye on his diabetes." | Both older adult and caregiver report diabetes | Diabetes | Diabetes | Older adult: because of a family history of complications from diabetes.  Caregiver: due to the short- and long-term effects on the small and large blood vessels, which could compromise the older adult’s independence and daily life | Higher risk of complications | Higher risk of complications | Agreement on chronic disease considered a priority and reason |
| 38  Mother & daughter | "Diabetes brings a lot of things. Going into a diabetic coma is no joke, it can happen just like that."  "What bothers me the most? My ear. It annoys me, you know, because it's not pleasant when I don’t understand and have to ask to repeat something. That’s the only thing.” | "For me, it’s diabetes and heart disease. Because if the heart stops, everything stops, first and foremost. And diabetes because of all the consequences it can bring." | Both older adult and caregiver identify diabetes,  Caregiver adds heart disease while older adult hypoacusis | Diabetes, Hypoacusis | Diabetes, Heart Disease | Older adult identifies diabetes for its complications and adds hypoacusis due to the embarrassment it causes in social situations.  He doesn’t prioritize heart disease because he believes it is linked to aging.  Caregiver highlights both diabetes and heart disease for their potential risks, particularly cardiovascular problems | Higher risk of complications  Perceived social impact | Higher risk of complications | Agreement on one disease considered a priority and reason |

*Note.* COPD, Chronic Obstructive Pulmonary Disease; TIA, Transient Ischemic Attack.
